# Low Back Pain and Disabilities Among Postpartum Women: Prevalence, Severity and Associated Factors

**DOI:** 10.3390/healthcare14070959

**Published:** 2026-04-06

**Authors:** Samiah Alqabbani, Maha F. Algabbani, Abeer A. Alazmi, Samiha M. I. Abdelkader, Mai Aldera, Lolwah AlRashed AlHumaid, Rehab F. M. Gwada, Munera M. Almurdi, Wafa Alahmari, Afrah Almuwais, Madawi Alotaibi, Jawahr Alagil, Afaf A. M. Shaheen

**Affiliations:** 1 Department of Rehabilitation Sciences, College of Health and Rehabilitation Sciences, Princess Nourah Bint Abdulrahman University, Riyadh 11671, Saudi Arabia; sfalqabbani@pnu.edu.sa (S.A.);; 2 Department of Rehabilitation Health Sciences, College of Applied Medical Sciences, King Saud University, Riyadh 11433, Saudi Arabia; 3 Department of Community Health Sciences, College of Applied Medical Sciences, King Saud University, Riyadh 11451, Saudi Arabia; 4 Physical Therapy Department, National Heart Institute, Giza 12613, Egypt; 5 Basic Sciences Department, Faculty of Physical Therapy, Cairo University, Cairo 12613, Egypt

**Keywords:** disability, musculoskeletal pain, post-partum, nordic musculoskeletal symptoms questionnaire, pain intensity

## Abstract

**Background**: Low back pain is a common musculoskeletal complaint among postpartum women due to physical changes that occur during pregnancy and delivery, which can lead to different disability levels. Therefore, the aim of this study was to evaluate the disability levels and associated factors of postpartum women within the first year after childbirth. **Methods**: A descriptive cross-sectional study design was used to gather data from post-partum women between 6 weeks and 12 months after childbirth using an online self-administered questionnaire. This questionnaire included demographic variables, the Nordic Musculoskeletal Symptoms Questionnaire, the Pain Intensity Numeric Rating Scale, and a back disability questionnaire. **Results**: Among 400 postpartum mothers, 71% reported low back pain, with 51.1% experiencing mild disability. Logistic regression showed significant predictors of disability, including cesarean delivery (6.49 times higher likelihood), having 4–5 children (1.98 times), and more than six children (3.45 times). Breastfeeding increased disability risk (2.44 times), while mixed feeding reduced it (0.52 times). The model explained 49.8% of disability variance (*p* < 0.001). **Conclusions**: Disability is a common problem among postpartum women, highlighting the importance of healthcare providers addressing these challenges.

## 1. Introduction

Low back pain (LBP) is among the most common musculoskeletal conditions globally, defined as pain localized between the lower rib margins and the gluteal folds, with or without leg pain, and is often presenting as non-specific with an unclear etiology [1]. It represents a significant health and economic burden, as it is the leading cause of disability and work absence worldwide and ranks sixth in overall disease burden [2].

The prevalence of LBP varies across populations, with certain groups experiencing higher rates. For example, individuals engaged in physically demanding occupations are at increased risk [3]. Gender differences also play a role, as ergonomic challenges and occupational demands have been identified as key contributors to LBP among women [4].

Among women, the postpartum period represents a particularly vulnerable time for developing musculoskeletal pain, including LBP. Postpartum women undergo significant physical, hormonal, and psychological changes that can contribute to musculoskeletal pain in various body regions [5,6,7]. Notably, this period extends beyond the immediate weeks after childbirth, with associated physical and mental health disorders potentially persisting for up to 12 months [8]. Research has shown that postpartum musculoskeletal pain can significantly affect a woman’s ability to care for her infant, further highlighting the need to understand LBP in this population [9,10].

Although LBP is commonly reported during pregnancy, the trajectory of back pain and associated disability throughout the extended postpartum period remains underexplored [11]. Some studies have examined the persistence of LBP after childbirth. For instance, Madzivhandila et al. (2023) reported that pregnancy-related LBP persisted in 44% of women at six weeks postpartum [12]. Tavares et al. (2020) found that 15–21% of women continued to experience LBP up to three months postpartum [13]. Additionally, Asif et al. (2022) reported a prevalence rate of 67.23% in 119 postpartum women up to six months after childbirth [14].

While previous studies have provided valuable insights into low back pain during the early postpartum period, most have focused on short-term outcomes, typically within the first six months after childbirth. However, the postpartum period may extend up to 12 months [15], during which women continue to experience ongoing physical and functional challenges. Despite this, there is limited evidence describing the overall burden of low back pain and associated disability across the extended postpartum period, particularly in diverse populations. In Saudi Arabia, postpartum women may be influenced by unique cultural, lifestyle, and healthcare-related factors, including variations in physical activity levels, childcare practices, and postpartum care utilization, which may affect the experience and impact of low back pain and disability. However, evidence from this population remains scarce. Understanding LBP prevalence and disability throughout this entire period is crucial for developing appropriate interventions.

Moreover, LBP is not only a source of pain but also a leading cause of disability worldwide, contributing significantly to long-term impairments in physical and mental functions [2]. Pain-related disability restricts individuals from performing daily activities and participating fully in their environment [16,17]. In postpartum women, persistent musculoskeletal pain is associated with long-term disability, with factors such as age and muscle endurance identified as key predictors following pelvic girdle pain [8]. Despite these findings, there remains limited research assessing the extent of back pain-related disability and its contributing factors in postpartum populations.

To the best of our knowledge, no previous studies have examined the prevalence of LBP and its associated disability among postpartum women in Saudi Arabia. Existing research has primarily focused on the early postpartum period or extended only up to six months postpartum. Considering that the postpartum period can last up to 12 months [15], this study aims to evaluate the prevalence, severity, and associated disability of low back pain among postpartum women within the first year after childbirth. Additionally, the study seeks to identify key factors influencing pain and disability levels during this extended period, thereby providing a more comprehensive understanding of LBP’s impact on postpartum women in this population.

## 2. Materials and Methods

### 2.1. Study Design

A descriptive cross-sectional study employing an online, self-administered questionnaire.

### 2.2. Setting

The questionnaires were distributed and collected from October to December 2022. Recruitment of participants occurred at three major hospitals—King Fahd Medical City, Prince Muhammad bin Abdulaziz Hospital, and Al-Yamamah Hospital—as well as at 48 primary care centers within the Riyadh Second Health Cluster in Saudi Arabia.

### 2.3. Participants

Participants were postpartum women within 6 weeks to 12 months after childbirth, with eligibility determined based on predefined inclusion and exclusion criteria.

#### Inclusion and Exclusion Criteria

The inclusion criteria included postpartum mothers aged 20 to 50 years, who were between six weeks and one year following childbirth. Exclusion criteria included mothers with orthopedic or neurological conditions including participants with underlying conditions that may cause low back pain (e.g., musculoskeletal deformities, vertebral fracture, malignancy, or infection) [3], those with preterm infants or infants with congenital anomalies, and those experiencing severe postpartum complications such as postpartum hemorrhage, severe infections (e.g., endometritis), deep vein thrombosis, or postpartum cardiomyopathy. Additionally, mothers who could not read or understand the Arabic language or those with cognitive limitations that would prevent them from accurately completing the questionnaires were excluded.

### 2.4. Sample Size

The sample size was determined as descried by Pourhoseingholi et al., 2013, using the formula: n = Z^2^ P (1 − P)/d^2^ [18]. Based on a power analysis, a minimum of 273 participants with postpartum LBP was required to ensure statistical validity.

### 2.5. Study Procedures

The questionnaire was evaluated for usability and technical performance by 10 postpartum mothers before its official distribution. The survey was subsequently sent via mobile numbers obtained from medical records to postpartum women who met the inclusion criteria. The online questionnaire was provided as an open-access link. Additionally, hospitals and healthcare centers displayed invitation barcodes for the survey in their waiting areas. To prevent duplicate responses, participants were asked to enter the last four digits of their mobile number.

### 2.6. Instrument

#### 2.6.1. Sociodemographic Characteristics

Information about participants’ general and maternal demographic characteristics was collected in the first section. Age, height, weight, educational level, employment status, and number of children were all included. The maternal characteristics included delivery mode, the presence of postpartum complications, whether the infant is preterm or has a congenital anomaly, and the type of feeding.

#### 2.6.2. Prevalence of Back Pain

The second section of the questionnaire collects information on the prevalence of back pain using the Nordic Musculoskeletal Questionnaire (NMQ) [19]. The NMQ is a widely used, standardized tool designed to assess musculoskeletal symptoms across different body regions, including the lower back. It focuses on the presence of pain or discomfort over specified time frames (such as the past 12 months and the past 7 days).

#### 2.6.3. Disability Level

The Oswestry Disability Index (ODI) was used to assess the level of disability among the study participants [20]. The ODI is designed to evaluate the degree of disability in individuals with low back pain. It contains 10 items assessing various activities of daily living, each scored from 0 to 5. The total score is expressed as a percentage, with higher scores corresponding to greater disability. The interpretation of the scores is as follows: 0–20% indicates minimal disability; 21–40% indicates moderate disability; 41–60% indicates severe disability; 61–80% indicates crippled; and scores over 80% suggest the individual is either bedridden or exaggerating their symptoms. The Arabic version was used as it demonstrated good reliability and validity [21].

#### 2.6.4. Pain Numeric Rating Scale

The Pain Numeric Rating Scale (PNRS) was used to assess the intensity of back pain. Participants were asked to rank their pain intensity from 0 to 10, with 10 representing the maximum unbearable pain and 0 representing no pain. Scores of 5 or less were interpreted as mild pain, scores of 6–7 as moderate pain, and scores of 8 or higher as severe pain [22,23]. The PNRS has shown good responsiveness in subjects with low back pain [24].

### 2.7. Ethical Approval

Ethical approval was granted by the Institutional Review Board of King Fahad Medical City (IRB Log Number: 22-427E) and with the 1964 Helsinki Declaration and its later amendments or comparable ethical standards. The survey included information about the study’s purpose, the estimated time required to complete the online questionnaire, and the contact details of the principal investigator. Participants’ privacy was ensured, with no personal identifiers or names disclosed. A consent statement was provided for participants to agree to take part before beginning the questionnaire.

### 2.8. Statistical Analysis

The analysis was conducted using IBM SPSS Statistics for Windows (Version 29. Armonk, NY, USA). The Shapiro–Wilk test was applied to examine the normality of the continuous variables (height, weight, and Arabic ODI). For continuous variables, descriptive statistics are calculated as mean and standard deviation (or median and first and third interquartile; IQ if skewed). Participants’ characteristics, pain intensity categories, and disability levels were described using frequency and percentage.

An analysis of cross-tabulation was conducted to examine the relationship between dependent variables (levels of disability expressed as ordinal variables), and independent variables. Disability level was correlated with age, BMI category, pain intensity, educational level, and number of children based on Kendall’s coefficient (τ^b^). Correlation coefficients were interpreted as follows: weak = 0.1–0.29; moderate = 0.3–0.49; and strong = 0.5–0.69 and very strong 0.7–1.

In the case of categorical variables, such as work status, mode of delivery, and type of feeding, Pearson’s chi-square test (X^2^) was used, and the effect size was determined by the Cramer’s V (φ^c^). In general, the φ^c^ was interpreted as follows: less than or equal to 0.04 = no or very weak, 0.05–0.09 = weak, 0.10–0.14 = moderate, 0.15–0.24 = strong, and larger than 0.25 = very strong. Fisher’s Exact Test was used when some of the expected cell counts were less than 5. 

To assess the presence of disability, participants were categorized into two groups: “no disability” and “disability present”. A binary logistic regression model was employed to determine the odds ratios associated with disability. Variables that demonstrated a significant correlation were included in the logistic regression analysis to identify potential predictors of disability.

## 3. Results

### 3.1. Participant’s Characteristics

**Figure 1:**
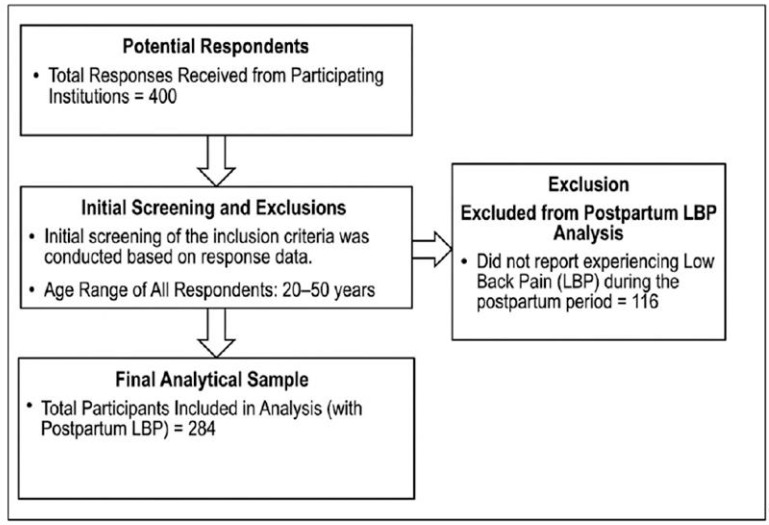
Flowchart of participant recruitment and selection process.

A total of 400 responses were received. Out of these responses 284 (71%) revealed that they complain from LBP during the postpartum period (Figure 1).

Participants ranged in age from 20 to 50 years (130; 45.8% were between 31 and 40 years), with the median (1st, 3rd IQ) being 160 cm (156, 164), 70 kg (60, 79), and 26.62 BMI (24.15, 29.74). The pain intensity ranged from mild pain to sever pain with a median (1st, 3rd IQ) of 5 (4, 7) and more than 50% complained from mild pain Table 1.

### 3.2. Prevalence of Pain and Disability

The prevalence of LBP during the postpartum period in the participants was 71% (284). Regarding pain intensity among participants with low back pain, more than half reported mild pain (57.7%), while 22.9% reported severe pain and 19.4% reported moderate pain. For Disability, more than half of participants with low back pain reported a mild disability level (145, 51.1%), while 93 (32.7%) had no disability, 42 (14.8%) reported a moderate disability, and 4 (1.4%) reported a severe to complete disability.

### 3.3. Correlation of Disability Level with the Independent Variables

Table 2 presents the results of cross-tabulation tests showed a positive weak significant association between disability category and the number of children, as well as the intensity of pain. As the number of children increases and pain intensity increases, disability increases. Additionally, disability was associated with work status, mode of delivery with a very strong effect size (φ^c^ = 0.26 and 0.29, respectively), and feeding type with strong effects (φ^c^ = 0.22). There was a higher percentage of participants who were not employed (70.1%) who reported a certain level of disability compared with working participants (64.3%) and a higher percentage of mothers who reported no disability in working mothers compared to non-employed mothers (33.9% and 29.9%, respectively).

Cesarean section mothers reported more disability than vaginal delivery mothers (82.2% and 57.5%, respectively). Additionally, mothers who mixed feed their babies (breastfeeding and formula feeding) reported a higher percentage of disabilities (75.1%) than mothers who exclusively breastfed (41.1%) or exclusively used formula (47.8%). Other independent variables did not show any associations with disability.

### 3.4. Regression Analysis

A logistic regression was performed to ascertain the effects of work status, number of children, mode of delivery, type of feeding and low back pain intercity on the likelihood that participants have any level of disability. The logistic regression model was statistically significant, χ2(9) = 125.74, *p* < 0.001. The model explained 49.8% (Nagelkerke R^2^) of the variance in disability and correctly classified 83.5% of cases.

Independent variables contributed significantly to the model (*p* < 0.05), except pain intensity (*p* = 0.12) did not (Table 3).

There was a reduction in disability likelihood associated with employment. The odds of having a disability were 6.49 times greater for mothers who had C-section deliveries. In contrast to mothers with no or one child, mothers with 4–5 children and more than six children had an increased likelihood of exhibiting disability by 1.98 times and 3.45 times, respectively. As compared to mothers who only use formula to feed their children, breastfeeding mothers were more likely to have disability (2.44 times) while mothers who mix formula and breastmilk were less likely to have disability (0.52 times).

## 4. Discussion

This study investigated low back pain and its associated levels of disability among postpartum women within the first year after childbirth. The results revealed that a significant proportion of women reported experiencing low back pain, with varying degrees of associated disability. Additionally, disability was significantly associated with several maternal and clinical factors, including number of children, employment status, mode of delivery, and type of feeding.

A high percentage of participants in our study experienced LBP during the first year postpartum, with 71% reporting this issue. Among these women, more than half (57.7%) reported mild pain intensity, while 19.4% experienced moderate pain, and 22.9% reported severe pain. These findings are consistent with those of Chungade et al. (2020), who identified low back pain as the most frequently reported musculoskeletal complaint, affecting 74% of cases [9]. Similarly, Asif et al. (2022) found LBP prevalence among 67.23% of postpartum women [14]. Additionally, the perceived back pain intensity in our sample (NPS: 5/10) was higher than those reported by Chungade et al. (NPS: 3.6/10) [9]. This discrepancy may be due to differences in population characteristics, as our sample included women in a higher age range with greater average BMI.

Further supporting these findings, other research has documented varying rates of persistent pregnancy-related LBP extending into the postpartum period. Studies report that LBP persists in a significant portion of women, with prevalence rates ranging from 15 to 21% within the first three months postpartum to as high as 67% up to six months postpartum, with nearly 44% experiencing continued pain at six weeks postpartum [12,13,14]. Notably, our study did not examine the history of back pain during pregnancy, which may have contributed to the persistence of pain in the postpartum period. These variations underscore the prolonged impact of LBP on postpartum women and emphasize the need for ongoing management and support throughout the postpartum period.

A varying level of disability due to low back pain was reported in 67.3% of participants, as measured by the Oswestry Disability Index, indicating that low back pain in postpartum women significantly impacts daily activities and participation. Among those who experienced disability, the severity levels varied: more than half (51.1%) reported mild disability, 14.8% experienced a moderate level, and only a small percentage (1.4%) faced severe or completely disabling conditions. These findings highlight the diverse impact of low back pain on postpartum women’s functionality and underscore the importance of addressing different levels of disability.

Studies investigating the extent of disability due to low back pain in postpartum women are limited, making direct comparison with similar studies challenging. However, our study identified an association between pain intensity and disability level, with higher pain intensity linked to greater disability. Evidence from broader low back pain populations supports this finding, with Canli and Özüdoğru (2026) identifying pain intensity as a significant predictor of disability, suggesting a consistent relationship between pain severity and functional limitation [25]. Additionally, the number of children was found to be associated with increased pain and disability levels.

These results are consistent with findings from a retrospective study by Zhang et al. (2023), which examined the association between childbirth and functional limitations due to low back pain in women [26]. Their study demonstrated that women with previous childbirth experience are more susceptible to developing disabling back pain compared to those who have not given birth, and that women who experience back pain during the postpartum stage are more likely to report severe back pain in later stages. Moreover, Heuch et al. 2020, indicated that the number of children is associated with chronic LBP [27].

In addition to the number of children, several other factors have emerged as correlates of low back pain and associated disability among postpartum women, including mode of delivery, work status, and type of feeding. Our study found that participants who delivered via Cesarean section reported a higher prevalence of disability (82.2%) compared to those who had vaginal deliveries (57.5%). This finding is consistent with previous research which also observed a correlation between mode of delivery and the prevalence of low back pain, although they did not assess its specific impact on disability levels [14]. Furthermore, a systematic review by Christopher et al. (2019) identified Cesarean section delivery as a significant factor contributing to the development of lumbopelvic pain in the postpartum period [28].

Additionally, this study demonstrated a relatively high odds ratio (OR = 6.49) for disability in the regression analysis, indicating a strong association between Cesarean delivery and disability. Similarly, Jin et al. reported an association between cesarean section and reduced activity levels [29]. Several mechanisms may explain this relationship, including surgical trauma, increased postoperative pain, reduced mobility during recovery, and impaired core muscle function following abdominal incision, all of which may contribute to higher levels of disability [30,31,32]. This evidence underscores the need for tailored postpartum support based on delivery methods to mitigate the risk of disability due to low back pain.

Feeding style also emerged as a significant factor in both our study and in the study by Asif et al. [14]. While Asif’s research reported that breastfeeding was associated with a higher prevalence of low back pain, our findings indicated that the type of feeding modality had a notable impact on disability levels [14]. Specifically, mothers who practiced both breastfeeding and bottle feeding reported the highest levels of disability (75.1%), compared to those who exclusively breastfed (41.1%) or exclusively used formula (47.8%). This pattern may reflect the increased physical demands associated with mixed feeding, which often requires prolonged static positions and frequent postural adjustments during infant care. In this context, postural factors may represent an important contributor to disability, as such sustained and repetitive loading may increase mechanical strain on the spine. This interpretation is supported by evidence from different populations indicating that posture is a significant predictor of disability in individuals with low back pain [25], and may help explain the higher disability levels observed in the present study. Moreover, research suggests that mothers who exclusively breastfeed tend to experience longer hours of night sleep, which may help mitigate musculoskeletal pain [33].

This alignment with prior research highlights the importance of further investigation into the risk factors contributing to low back pain and disability in postpartum women. Collectively, this body of evidence underscores the need for targeted postpartum support and ergonomic education to reduce the risk of disability associated with mode of delivery, feeding practices, and other influencing factors. Supporting this, Li et al. (2024) found that mothers who received support during the postpartum period experienced decreased muscular strain, ultimately leading to lower pain levels associated with postpartum recovery [10]. This evidence underscores the need for tailored postpartum support based on delivery methods to mitigate the risk of disability due to low back pain.

From a contextual perspective, several cultural and lifestyle-related factors may influence the experience of low back pain and associated disability among postpartum women in Saudi Arabia. These may include variations in physical activity levels and postpartum recovery practices that may involve periods of reduced mobility [34]. In addition, caregiving responsibilities, including infant feeding practices, may contribute to physical strain. In the Saudi context, breastfeeding-related activities have been associated with musculoskeletal pain among lactating mothers [35], which may reflect the impact of sustained postures during infant care. Although these factors were not directly assessed in the present study, they may provide additional context for interpreting the findings.

In Saudi Arabia, postpartum care is guided by recommended follow-up protocols; however, available evidence suggests that attendance at postpartum visits may not be optimal [36]. In addition, these visits typically focus on general maternal and neonatal health, such as breastfeeding support and contraception counseling, while musculoskeletal conditions such as low back pain are not routinely addressed [37]. Given the high prevalence of low back pain and associated disability observed in the present study, incorporating targeted screening and appropriate referral pathways into postpartum care may help improve maternal functional outcomes.

This study encountered several limitations. First, the cross-sectional design does not allow causal relationships to be established between the studied variables. Second, the data were collected using a self-administered questionnaire, which may be subject to recall bias and variability in participants’ understanding of the questions. In addition, low back pain was identified based on self-report without clinical confirmation by a healthcare professional, which may affect diagnostic accuracy. Third, the history of low back pain during pregnancy was not assessed, which may have influenced postpartum pain and disability levels. Finally, the use of convenience sampling may limit the generalizability of the findings. Future research should focus on strategies to address factors contributing to higher disability levels in postpartum women and explore targeted interventions to support mothers, minimizing the impact of disability on their daily lives and overall well-being. In addition, longitudinal studies incorporating clinical assessment, objective functional measures, and psychosocial factors are needed to provide a more comprehensive understanding of disability in this population.

## 5. Conclusions

This study highlights the high prevalence of low back pain among postpartum women, which is often accompanied by varying degrees of disability. Factors associated with an increased risk of disability in one year postpartum include a higher number of children, Cesarean section delivery, employment status, and combining breastfeeding with bottle feeding. These findings underscore the importance of addressing these specific risk factors in postpartum care to reduce the impact of low back pain and associated disability on women’s health and daily functioning.

## Figures and Tables

**Table 1:** Demographic characteristic of participants.

Participant’s Characteristics		Participants with LBP (Number of Participants = 284)
		Frequency (Percentage)
Age group	20–30	99 (34.9%)
	31–40	130 (45.8%)
	41–50	55 (19.4%)
BMI classification	Healthy	96 (33.8%)
	Overweight	119 (41.9%)
	Obese	69 (24.3%)
Education level	High school or lower	90 (31.7%)
	Bachelor’s degree or higher	194 (68.3%)
Work status	Working	137 (48.2%)
	Not working	147 (51.8%)
Number of children	0–1	32 (11.3%)
	2–3	148 (52.1%)
	4–5	69 (24.3%)
	More than 6	35 (12.3%)
Mode of delivery	Spontaneous Vaginal Delivery	172 (60.6%)
	Cesarean section	112 (39.4%)
Type of feeding	Exclusive breastfeeding	34 (12.0%)
	Exclusive baby formula	41 (14.4%)
	Mixed type	209 (73.6%)
LBP intensity	Mild	164 (57.7%)
	Moderate	55 (19.4%)
	Sever	65 (22.9%)

**Table 2:** Correlation of disability with participants’ characteristics.

Variables	Disability Category (Number of Participants = 284)				Cross-Tabulation Tests	
	No	Mild	Moderate	Sever or Complete	Value ^†^	*p*
Age group						
*20–30*	30 (30.3%)	62 (62.6%)	5 (5.1%)	2 (2.0%)	0.04 (0.05)	0.44
*31–40*	44 (33.8%)	58 (44.6%)	27 (20.8%)	1 (0.8%)		
*41–50*	19 (34.5%)	25 (45.5%)	10 (18.2%)	1 (1.8%)		
BMI						
*Healthy*	23 (24.0%)	67 (69.8%)	3 (3.1%)	3 (3.1%)	−0.05 (0.05)	0.40
*Overweight*	40 (33.6%)	52 (43.7%)	26 (21.8%)	1 (18.8%)		
*Obese*	23 (33.7%)	35 (50.7%)	10 (14.9)	1 (1.4%)		
Education						
*High school or lower*	32 (35.6%)	31 (34.4%)	26 (28.9%)	1 (1.1%)	−0.08 (0.06)	0.14
*Bachelor’s or higher*	61 (31.4%)	114 (58.8%)	16 (8.2%)	3 (1.5%)		
Work status						
*Employed*	49 (35.8%)	79 (57.7%)	7 (5.1%)	2 (1.5%)	19.77 (3)	0.01
*Non-Employed*	44 (29.9%)	66 (44.9%)	35 (23.8%)	2 (1.4%)		
Children number						
*0–1*	17 (53.1%)	13 (40.6%)	1 (3.1%)	1 (3.1%)	0.21 (0.05)	<0.001
*2–3*	51 (34.5%)	80 (54.1%)	14 (9.5%)	3 (2.0%)		
*4–5*	20 (29.0%)	33 (47.8%)	16 (23.2%)	0		
*More than 6*	5 (14.3%)	19 (54.3%)	11 (31.4%)	0		
Mode of delivery						
*Vaginal*	73 (42.4%)	69 (40.1%)	27 (15.7%)	3 (1.7%)	23.33 (3)	<0.001
*C-section*	20 (17.9%)	76 (67.9%)	15 (13.4%)	1 (0.9%)		
Feeding type						
*formula*	21 (51.2%)	19 (46.3%)	1 (2.4%)	0	28.49 (6)	<0.001
*breastfeeding*	20 (58.8%)	13 (38.2%)	1 (2.9%)	0		
*Mixed type*	52 (24.9%)	113 (54.1%)	40 (19.1%)	4 (1.9%)		
Pain intensity						
*Mild*	75 (45.7%)	60 (36.6%)	28 (17.1%)	1 (0.6%)	0.25 (0.05)	<0.001
*Moderate*	18 (32.7%)	33 (60%)	3 (5.5%)	1 (1.8%)		
*Sever*	0	52 (80.0%)	11 (16.9%)	2 (3.1%)		

^†^ Kendall’s coefficient τ^b^ (standard error) was used for ordinal variables such as age, BMI, educational level, and number of children, and Pearson’s chi-square test X^2^ (degree of freedom) was used for other categorical variables. Fisher’s Exact Test Fisher’s Exact Test was used when some of the expected cell counts were less than 5. *p* level of significant <0.05.

**Table 3:** Logistic regression models for back disability correlation.

Characteristic (Number of Participants = 284)		B	SE	Wald	df	*p*	Odd Ratio	95% CI	
								Lower Bound	Upper Bound
Work status	Non-Employed (Reference category)								
	*Employed*	−0.925	0.356	6.735	1	0.009	0.397	0.197	0.797
Mode of delivery	Vaginal (Reference category)								
	Cesarean section	1.870	0.359	27.111	1	<0.001	6.487	3.209	13.112
Number of children	0–1 (Reference category)								
	*2–3*	−0.512	0.653	0.616	1	0.432	0.599	0.167	2.153
	*4–5*	0.685	0.700	0.958	1	0.328	1.983	0.503	7.813
	*More than 6*	1.238	0.854	2.101	1	0.147	3.450	0.647	18.410
Feeding type	formula (Reference category)								
	breastfeeding	0.890	0.542	2.693	1	0.101	2.435	0.841	7.051
	Mixed type	−0.651	0.679	0.920	1	0.337	0.521	0.138	1.973

B = beta; SE = stander of error, df = degree of freedom, CI = confidence interval; *p* = Level of significance, α = 0.05.

## Data Availability

The datasets generated and/or analyzed during the current study are not publicly available due to ethical and privacy restrictions, but are available from the corresponding author on reasonable request.
